# Classification of quaternionic skew-Hermitian symmetric spaces

**DOI:** 10.1007/s00605-026-02225-y

**Published:** 2026-09-30

**Authors:** Ioannis Chrysikos, Jan Gregorovič

**Affiliations:** 1 Department of Mathematics and Statistics, Faculty of Science, Masaryk University, Kotlářská 2, 611 37 Brno, Czech Republic; 2 Department of Mathematics, Faculty of Science, University of Ostrava, 701 03 Ostrava, Czech Republic; 3 Institute of Discrete Mathematics and Geometry, TU Vienna, Wiedner Hauptstrasse 8-10/104, 1040 Vienna, Austria

**Keywords:** Homogeneous spaces, Quaternionic skew-Hermitian symmetric spaces, Involutive Lie algebras, Classification, 53C10, 53C15, 53C30, 53C35

## Abstract

We provide a complete classification of quaternionic skew-Hermitian symmetric spaces, namely symmetric spaces that admit a torsion-free ${{\,\mathrm{\textsf{SO}}\,}}^*(2n){{\,\mathrm{\textsf{Sp}}\,}}(1)$-structure for arbitrary n>1. Moreover, we prove that any homogeneous quaternionic skew-Hermitian manifold is necessarily a symmetric space.

## Introduction

This work is devoted to the classification of simply connected symmetric spaces admitting an invariant ${{\,\mathrm{\textsf{SO}}\,}}^*(2n){{\,\mathrm{\textsf{Sp}}\,}}(1)$-structure, known as quaternionic skew-Hermitian symmetric spaces (briefly, qs-H symmetric spaces). Recall by [7] that an ${{\,\mathrm{\textsf{SO}}\,}}^*(2n){{\,\mathrm{\textsf{Sp}}\,}}(1)$-structure on a 4*n*-dimensional manifold *M* with quaternionic dimension n>1 is determined by a pair (Q,ω), where $Q\subset {{\,\mathrm{\textsf{End}}\,}}(TM)$ is an almost quaternionic structure on *M* and $\omega \in \Lambda ^2T^*M$ is a non-degenerate 2-form which is *Q*-Hermitian, usually referred to as a scalar 2-form. Manifolds admitting such a $\textsf{G}$-structure are called almost quaternionic skew-Hermitian manifolds, abbreviated as almost qs-H manifolds. In the torsion-free (i.e., 1-integrable) case the prefix “almost” is omitted and one speaks of quaternionic skew-Hermitian structures, abbreviated as qs-H structures. A quaternionic skew-Hermitian symmetric space is then defined as a symmetric space admitting a quaternionic skew-Hermitian structure (Q,ω) such that each symmetry is a quaternionic skew-Hermitian automorphism of (Q,ω). Here, by the term quaternionic skew-Hermitian automorphism (in short, qs-H automorphism) we mean a diffeomorphism preserving both *Q* and ω.

In this work we show that these symmetric spaces are the only homogeneous qs-H manifolds.

### Theorem 1.1

Any homogeneous qs-H manifold is a qs-H symmetric space.

Moreover, we prove the following classification theorem:

### Theorem 1.2

Let *M* be a qs-Hermitian symmetric space. Then, up to covering, *M* is one of the following spaces:

(1) The linear model $\mathbb {H}^n\cong [{{\,\mathrm{\textsf{E}}\,}}{{\,\mathrm{\textsf{H}}\,}}]$ (see [7, Sect. 2] for details on the linear model).

(2) One of the simple symmetric spaces:

$$\begin{aligned} M_{1}&={{\,\mathrm{\textsf{SO}}\,}}^*(2n+2)/{{\,\mathrm{\textsf{SO}}\,}}^*(2n)\textsf{U}(1)\,,\\ M_{2}&={{\,\mathrm{\textsf{SU}}\,}}(2+p,q)/({{\,\mathrm{\textsf{SU}}\,}}(2){{\,\mathrm{\textsf{SU}}\,}}(p,q)\textsf{U}(1))\,,\\ M_{3}&={{\,\mathrm{\textsf{SL}}\,}}(n+1,\mathbb {H})/({{\,\mathrm{\textsf{GL}}\,}}(1,\mathbb {H}){{\,\mathrm{\textsf{SL}}\,}}(n,\mathbb {H}))\,. \end{aligned}$$

(3) A non-semismple symmetric space $M=\textsf{G}/\textsf{L}$ where $\textsf{G}$ and $\textsf{L}$ have the Levi decompositions

$$ \textsf{G}={{\,\mathrm{\textsf{Sp}}\,}}(p+1, q)\ltimes \textsf{R}\,,\quad \textsf{L}=({{\,\mathrm{\textsf{Sp}}\,}}(1){{\,\mathrm{\textsf{Sp}}\,}}(p, q))\ltimes \textsf{K}$$

for solvable groups $\textsf{R}$ and $\textsf{K}$, respectively, and the Lie algebras $\mathfrak {r}$ and $\mathfrak {k}=\mathfrak {r}\cap \mathfrak {l}$ of the groups $\textsf{R}$ and $\textsf{K}$ are specified according to whether the quaternionic dimension *n* of *M* is even or odd:

${\text{( }3a)}$: For *n* even, such symmetric spaces $\textsf{G}/\textsf{L}$ are classified by integer parameters *p*, *q* satisfying $0\le p, q\le \frac{n}{2}$ and $0<p+q \le \frac{n}{2}$, see Example 4.3.
${\text{( }3b)}$: For *n* odd, such symmetric spaces $\textsf{G}/\textsf{L}$ are classified by integer parameters *p*, *q* satisfying $0\le p, q\le \frac{n-1}{2}$ and $0<p+q \le \frac{n-1}{2}$, see Example 4.4.

For both these classes the quaternionic pseudo-Kähler symmetric space ${{\,\mathrm{\textsf{Sp}}\,}}(p+1, q)/({{\,\mathrm{\textsf{Sp}}\,}}(1){{\,\mathrm{\textsf{Sp}}\,}}(p, q))$ is the semisimple factor of $\textsf{G}/\textsf{L}$.

Note that the semisimple qs-H symmetric spaces were previously classified in [7], building on the classification results of [1, 12]. The method introduced in the present work recovers the classification of the semisimple case and thus provides an alternative proof. Moreover, it extends beyond the semisimple setting and yields the full classification. It combines a construction of local torsion-free ${{\,\mathrm{\textsf{SO}}\,}}^*(2n){{\,\mathrm{\textsf{Sp}}\,}}(1)$-structures, derived by Cahen and Schwachhöfer [4] (see also [7, Proposition 6.3]), and the classification of normal forms of elements of the Lie algebra $\mathfrak {so}^*(2n)\oplus \mathfrak {sp}(1)$, contained in [10].

The organization of the paper goes as follows. In Sect. 2 we review necessary preliminaries on symmetric spaces. In Sect. 3 we describe the algebraic structure equivalent to a qs-H symmetric space, by introducing the notion of quaternionic skew-Hermitian transvection involutive Lie algebras. In Sect. 4 we determine when the Cahen-Schwachhöfer construction yields a qs-H symmetric space, see Proposition 4.2. Then Theorem 1.2, that is, the full classification of qs-H symmetric spaces (up to covering) is obtained as a combination of Theorem 4.8 and a basic correspondence obtained in terms of quaternionic skew-Hermitian transvection involutive Lie algebras, see Theorem 3.4, and see also Examples 4.3–4.7 for details. In Sect. 5 we prove Theorem 1.1 and, moreover, show that homogeneous almost qs-H structures do exist. In particular, the construction employed in this paper yields homogeneous almost qs-H manifolds of intrinsic torsion type $\mathcal {X}_4$ (conformally symplectic structures), see Example 5.2 (we refer to [7, 8] for the intrinsic torsion types of ${{\,\mathrm{\textsf{SO}}\,}}^*(2n){{\,\mathrm{\textsf{Sp}}\,}}(1)$-structures).

## The structure of quaternionic skew-Hermitian symmetric spaces

Our description begins with a brief summary of symmetric spaces, providing background relevant to the discussion that follows. There are many equivalent ways to define symmetric spaces. A review of these approaches is given in [11, Sect. 1.3] and further details can be found, for example, in [2, 13].

### Generalities on symmetric spaces

A symmetric space can be viewed as a pair (*M*, *s*) where *M* is a connected smooth manifold and $s : M\times M\rightarrow M$ is a smooth map such that the family

$$ \{s_x : M\rightarrow M\}_{x\in M} $$

where $s_{x}(y):=s(x, y)$ for all x,y∈M, is a (smooth) family of involutions of *M* satisfying the following properties:

1. $$s_{x}(x)=x$$ for all x∈M;
2. $$s_{x}\circ s_{y}\circ s_{x}=s_{s_{x}(y)}$$ for all x,y∈M;
3. For each x∈M there is a neighbourhood $U_{x}\subset M$ in which *x* is the only fixed point of $s_{x}$.

Each $s_{x} : M\rightarrow M$ is called the symmetry centered at *x* and by the term “involution” we mean that $s_{x}(s_{x}(y))=y$ for all x,y∈M. Note that the third axiom (*S*3) means that the point *x* is an isolated fixed point of $s_x$, for all x∈M. A morphism between two symmetric spaces (*A*, *s*) and (*B*, *t*), with smooth families of involutions $\{s_a\}_{a\in A}$ and $\{t_b\}_{b\in B}$, respectively, is a smooth map f:A→B with $f(s_{x}(y))=t_{f(x)}(f(y))$ for any x,y∈A. Such morphisms will be referred to as symmetric morphisms. The automorphism group of a symmetric space $(M, \{s_x\}_{x\in M})$ is the group of all symmetric automorphisms of *M*, denoted by ${{\,\mathrm{\textsf{Aut}}\,}}(M, s)$. By the second axion (*S*2) we see that any symmetry $s_x$ centered at x∈M is a symmetric automorphism of *M*, thus there is a natural inclusion of the group generated by the symmetries of (*M*, *s*) into ${{\,\mathrm{\textsf{Aut}}\,}}(M, s)$. The smallest subgroup of the group generated by the symmetries of a symmetric space (*M*, *s*) which acts transitively on *M* is the transvection group, denoted by $\textsf{Tr}(M, s):=\langle s_x\circ s_y : x, y\in M\rangle$.

For the moment, let us denote this group by $\textsf{G}=\textsf{Tr}(M, s)$ and fix a base point o∈M. Since $\textsf{G}$ is transitive we have a homogeneous presentation $M=\textsf{G}/\textsf{L}$, where $\textsf{L}=\{g\in \textsf{G}: go=o\}$ is the stabilizer of o∈M. Note that $\textsf{L}$ is a closed subgroup of $\textsf{G}$. Moreover, the rule $\sigma (g):=s_o\circ g\circ s_o$ for all $g\in \textsf{G}$, defines an involution $\sigma : \textsf{G}\rightarrow \textsf{G}$ of $\textsf{G}$ and the stabilizer $\textsf{L}$ satisfies $(\textsf{G}^{\sigma })^{0}\subset \textsf{L}\subset \textsf{G}^{\sigma }$, where $\textsf{G}^{\sigma }$ is the closed subgroup of $\textsf{G}$ consisting of elements fixed by σ and $(\textsf{G}^{\sigma })^{0}$ denotes its connected component. Conversely, a pair $(\textsf{G}, \sigma )$ of a connected Lie group $\textsf{G}$ endowed with a non-trivial involutive automorphism $\sigma : \textsf{G}\rightarrow \textsf{G}$ gives rise to a symmetric space $\textsf{G}/\textsf{L}$, where $\textsf{L}$ is any subgroup with $(\textsf{G}^{\sigma })^{0}\subset \textsf{L}\subset \textsf{G}^{\sigma }$. Often, such triples $(\textsf{G}, \sigma , \textsf{L})$ are referred to as symmetric pairs, see for example [2, p. 3].

#### Definition 2.1

A finite-dimensional transvection involutive Lie algebra (tiLa in short) consists of a finite-dimensional real Lie algebra $\mathfrak {g}$ and an involutive Lie algebra automorphism $\sigma : \mathfrak {g}\rightarrow \mathfrak {g}$ such that the following two condition are satisfied:

1. The $\mathbb {Z}_2$-grading of $\mathfrak {g}$, given by
  $$ \mathfrak {g}=\mathfrak {l}\oplus \mathfrak {m}\,, \quad \mathfrak {l}:=\{X\in \mathfrak {g} : \sigma (X)= X\}\,,\quad \mathfrak {m}:=\{X\in \mathfrak {g} : \sigma (X)=- X\}, $$
  satisfies the condition $\mathfrak {l}=[\mathfrak {m}, \mathfrak {m}]$.
2. No non-zero ideal of $\mathfrak {g}$ is contained in $\mathfrak {l}$.

Given a tiLa $(\mathfrak {g}, \sigma )$, the decomposition $\mathfrak {g}=\mathfrak {l}\oplus \mathfrak {m}$ into ±1-eigenspaces of σ is referred to as the canonical decomposition of $\mathfrak {g}$. Since this decomposition is a $\mathbb {Z}_2$-grading, the relations $[\mathfrak {l}, \mathfrak {l}]\subset \mathfrak {l}$ and $[\mathfrak {l}, \mathfrak {m}]\subset \mathfrak {m}$ hold as well. Thus $\mathfrak {l}$ is a Lie subalgebra of $\mathfrak {g}$ and $\mathfrak {m}$ is an $\mathfrak {l}$-module, which corresponds to a representation

$$ \rho : \mathfrak {l}\rightarrow {{\,\mathrm{\textsf{End}}\,}}(\mathfrak {m}) $$

defined by the restriction of the adjoint action. This is a faithful representation, as a consequence of the second condition in the Definition 2.1. Moreover we mention that the induced $\mathfrak {l}$-action on the endomorphism algebra ${{\,\mathrm{\textsf{End}}\,}}(\mathfrak {m})$ of $\mathfrak {m}$ can be interpreted as

$$ \rho (X)A:=[\rho (X), A] $$

for all $X\in \mathfrak {l}$ and $A\in {{\,\mathrm{\textsf{End}}\,}}(\mathfrak {m})$, where the bracket is the commutator of endomorphisms (extending the bracket on $\mathfrak {l}\subset \mathfrak {g}$).

Associated to any symmetric pair $(\textsf{G}, \sigma , \textsf{L})$, where $\textsf{G}$ is the transvection group, there is a unique tiLa given by $(\mathfrak {g}:={{\,\mathrm{\textsf{Lie}}\,}}(\textsf{G}), \sigma )$. Here, we maintain the same notation σ for the induced involution at the Lie algebra level, and, as usual, we denote by $\mathfrak {g}={{\,\mathrm{\textsf{Lie}}\,}}(\textsf{G})$ the Lie algebra of $\textsf{G}$.

#### Theorem 2.2

([2, 14]) There is a bijective correspondence between tiLas $(\mathfrak {g}, \sigma )$ and symmetric pairs $(\textsf{G}, \sigma , \textsf{L})$, where $\textsf{G}$ is the simply connected Lie group corresponding to the Lie algebra $\mathfrak {g}$ and $\textsf{L}$ is the closed connected subgroup of $\textsf{G}$ with Lie algebra $\mathfrak {l}$. In particular, there is a bijection between isomorphism classes of simply connected symmetric spaces and isomorphism classes of tiLas.

Recall that given a smooth connected manifold *M* with an affine connection ∇, the group $\textsf{Aff}(M, \nabla )$ of affine transformations of (M,∇) consists of diffeomorphisms $\varphi : M\rightarrow M$ preserving ∇, i.e., $\varphi ^*\nabla =\nabla$. An affine symmetric space is a connected manifold *M* endowed with a connection ∇ which satisfies the following property for each point x∈M: there is an involutive affine transformation $s_{x}\in \textsf{Aff}(M, \nabla )$ of (M,∇) such that *x* is an isolated point of $s_x$. Clearly, for each x∈M such an affine transformation $s_x$ is unique, and it turns out that an affine symmetric space (M,∇) is a symmetric space (*M*, *s*), as defined above. Conversely, any symmetric space is an affine symmetric space endowed with its unique canonical connection $\nabla ^{0}$ invariant under the symmetries. Moreover, $\nabla ^0$ is torsion-free, complete, and its curvature tensor $R^{0}$ is $\nabla ^{0}$-parallel, see [13].

### Quaternionic skew-Hermitian symmetric spaces

Symmetric spaces can be equipped with various geometric structures invariant with respect to the symmetries. For example, a pseudo-Riemannian symmetric space is a symmetric space $(M, \{s_x\}_{x\in M})$ endowed with a pseudo-Riemannian metric *g* such that each $s_x$ is an isometry of *g*, a complex symmetric space is a symmetric space endowed with a complex structure *J* such that each $s_x$ is a holomorphic map with respect to *J*, while a symplectic symmetric space is a symmetric space endowed with a symplectic 2-form ω such that each $s_x$ is a symplectic map with respect to ω. In this text we are interested in the following type of symmetric spaces.

#### Definition 2.3

A quaternionic skew-Hermitian symmetric space is a symmetric space $(M, \{s_x\}_{x\in M})$ such that each symmetry $s_x$ preserves a quaternionic structure *Q* on *M* and a symplectic 2-form ω on *M* which is *Q*-Hermitian.

Smooth 4*n*-dimensional manifolds *M* admitting an almost quaternionic structure $Q\subset {{\,\mathrm{\textsf{End}}\,}}(TM)$ and an almost symplectic form ω which is *Q*-Hermitian are called almost quaternionic skew-Hermitian manifolds (shortly, almost qs-H manifolds), denoted by $(M^{4n}, Q, \omega )$. The diffeomorphisms preserving *Q* and ω are called qs-H automorphisms and form a group, denoted by ${{\,\mathrm{\textsf{Aut}}\,}}(M, Q, \omega )$.

In terms of $\textsf{G}$-structures, such geometric structures correspond to a ${{\,\mathrm{\textsf{SO}}\,}}^*(2n){{\,\mathrm{\textsf{Sp}}\,}}(1)$-structure on *M*. We extensively studied these geometries in [7, 8], see also [9]. According to [7, Theorem 4.12], any almost qs-H manifold $(M^{4n}, Q, \omega )$ admits a unique minimal adapted ${{\,\mathrm{\textsf{SO}}\,}}^*(2n){{\,\mathrm{\textsf{Sp}}\,}}(1)$-connection, denoted by $\nabla ^{Q, \omega }$. It follows that the ${{\,\mathrm{\textsf{SO}}\,}}^*(2n){{\,\mathrm{\textsf{Sp}}\,}}(1)$-structure (Q,ω) is 1-integrable if and only if $\nabla ^{Q, \omega }$ is torsion-free, see [8, Proposition 2.9] for more details on the torsion-free case. The reason that we do *not* include the more general almost quaternionic or almost symplectic structures in the Definition 2.3 is explained, among other facts, in the following proposition.

#### Proposition 2.4

Let (*M*, *s*) be a symmetric space admitting an almost quaternionic skew-Hermitian structure (Q,ω) invariant under the symmetries. Then, the canonical connection $\nabla ^0$ has the following properties:

1. For all $X, Y, Z\in \mathfrak {X}(M)$ and x∈M we have $\omega _x(\nabla ^0_{X}Y, Z)=\frac{1}{2}X_{x}(\omega (Y+s_{x_{*}}Y, Z))$, where $s_{x_{*}}:={{\,\textrm{d}\,}}s_{x}$.
2. The connections $\nabla ^{0}$ and $\nabla ^{Q, \omega }$ coincide, $\nabla ^{0}=\nabla ^{Q, \omega }$ and hence the pair (Q,ω) is necessarily 1-integrable.
3. Any qs-H automorphism $f\in {{\,\mathrm{\textsf{Aut}}\,}}(M, Q, \omega )$ satisfies $f\circ s_{x}=s_{f(x)}\circ f$, for all x∈M.
4. The linear holonomy algebra of $\nabla ^{0}=\nabla ^{Q, \omega }$ coincides with the Lie subalgebra $\mathfrak {l}$, where $\mathfrak {g}=\mathfrak {l}\oplus \mathfrak {m}$ is the associated canonical decomposition, that is,
  $$ \mathfrak {l}\cong \textsf{hol}(\nabla ^{0})=\{R^{0}(X, Y)=-{{\,\textrm{ad}\,}}_{\mathfrak {m}}([X, Y]) : X, Y\in \mathfrak {m}\}\subset \mathfrak {so}^{*}(2n)\oplus \mathfrak {sp}(1)\,. $$

#### Proof

We recall that any qs-H transformation is affine with respect to the ${{\,\mathrm{\textsf{SO}}\,}}^*(2n){{\,\mathrm{\textsf{Sp}}\,}}(1)$-connection $\nabla ^{Q, \omega }$, see [7, Proposition 5.8]. Since $\nabla ^{Q, \omega }$ is unique and assumed that is invariant by the symmetries, this implies that the two connections $\nabla ^0$ and $\nabla ^{Q, \omega }$ coincide, $\nabla ^0=\nabla ^{Q, \omega }$. The second claim in (2) follows now since $\nabla ^0$ is torsion-free. Therefore ω is symplectic, and the expression given in (1) can be proved as in [3, Proposition A.1]. Part (3) follows easily by the fact that $\nabla ^0=\nabla ^{Q, \omega }$ is invariant under any qs-H automorphism $f\in {{\,\mathrm{\textsf{Aut}}\,}}(M, Q, \omega )$. Part (4) follows by Theorem 3.2 in [13, p. 232]. □

By part (3) of Proposition 2.4 we obtain

#### Corollary 2.5

The category of qs-H symmetric spaces with morphisms the qs-H transformations is a full subcategory of the category of qs-H manifolds, that is, ${{\,\mathrm{\textsf{Aut}}\,}}(M, Q, \omega )\subset {{\,\mathrm{\textsf{Aut}}\,}}(M, s)$.

## Quaternionic skew-Hermitian transvection involutive Lie algebras

In this section, we will establish a bijective correspondence between the isomorphism classes of simply connected quaternionic skew-Hermitian symmetric spaces and the isomorphism classes of the following purely Lie-algebraic object:

### Definition 3.1

A quaternionic skew-Hermitian transvection involutive Lie algebra (briefly, qs-H tiLa) is a quadruple $(\mathfrak {g}, \sigma , Q_0, \omega _0)$, where:

1. The pair $(\mathfrak {g}, \sigma )$ is a finite-dimensional transvection involutive Lie algebra.
2. The triple $(\mathfrak {m}, Q_0, \omega _0)$ is an $\mathfrak {l}$-invariant quaternionic skew-Hermitian vector space, where $\mathfrak {g}=\mathfrak {l}\oplus \mathfrak {m}$ is the associated canonical decomposition of $\mathfrak {g}$.

### Remark 3.2

Let $(\mathfrak {g}, \sigma , Q_0, \omega _0)$ be a qs-H tiLa with $\mathbb {Z}_2$-grading $\mathfrak {g}=\mathfrak {l}\oplus \mathfrak {m}$, as described above. Next we would like to remark a few important points:

(1) Since $(\mathfrak {g}, \sigma )$ is an involutive Lie algebra with canonical decomposition $\mathfrak {g}=\mathfrak {l}\oplus \mathfrak {m}$, we have $B(\mathfrak {l}, \mathfrak {m})=0$, where *B* is the Killing form $\mathfrak {g}$. If *B* is non-degenerate, so are its restrictions to $\mathfrak {l}$ and $\mathfrak {m}$, respectively, see [13, p. 233].

(2) By the second condition in Definition 3.1, we require that both the geometric objects $Q_0$ and $\omega _0$ are invariant under the action of the subalgebra $\mathfrak {l}=[\mathfrak {m}, \mathfrak {m}]$. In particular, saying that $Q_0\subset {{\,\mathrm{\textsf{End}}\,}}(\mathfrak {m})$ is $\mathfrak {l}$-invariant we mean that $[\rho (X), A]\in Q_0$ for any $X\in \mathfrak {l}$ and $A\in Q_0$. In analogy, $\omega _0 : \mathfrak {m}\times \mathfrak {m}\rightarrow \mathbb {R}$ is a linear scalar 2-form on $\mathfrak {m}$ with respect to $Q_0$, which is invariant under the action of $\mathfrak {l}$, that is, $\omega _0([X, U], W)+\omega _0(U, [X, W])=0$ for all $X\in \mathfrak {l}$ and $U, V\in \mathfrak {m}$.

(3) We can extend $\omega _0$ to the whole $\mathfrak {g}$ by requiring $X\lrcorner \omega _0=0$ for all $X\in \mathfrak {l}$, and denote the extension by the same symbol. By the $\mathfrak {l}$-invariance of $\omega _0$ and the relation $[\mathfrak {m},\mathfrak {m}]=\mathfrak {l}$, it is clear that $\omega _0$ is a Chevalley-Eilenberg 2-cocycle of the trivial representation of $\mathfrak {g}$ on $\mathbb {R}$. This means that $\omega _0$ belongs to the space

$$ Z^{2}(\mathfrak {g}, \mathbb {R})=\left\{ \alpha \in C^{2}(\mathfrak {g}, \mathbb {R})={{\,\mathrm{\textsf{Hom}}\,}}(\bigwedge ^{2}\mathfrak {g}, \mathbb {R}) : {{\,\textrm{d}\,}}\alpha =0\right\} $$

of Chevalley 2-cocycles of the trivial representation of $\mathfrak {g}$ on $\mathbb {R}$.

Let us now define the isomorphisms in the category of qs-H tiLas.

### Definition 3.3

Two qs-H tiLas $(\mathfrak {g}_{i}, \sigma _i, Q_i, \omega _i)$ (i=1,2), are said to be isomorphic if there exists a Lie algebra isomorphism $\varphi : \mathfrak {g}_1\rightarrow \mathfrak {g}_2$ such that $\varphi \circ \sigma _1 = \sigma _2 \circ \varphi$, $\varphi ^* Q_2 = Q_1$ and $\varphi ^* \omega _2 = \omega _1$.

### Theorem 3.4

There is a bijection between isomorphism classes of qs-H tiLas $(\mathfrak {g}, \sigma , Q_0, \omega _0)$ and isomorphism classes of simply connected qs-H symmetric spaces (M,Q,ω,s).

### Proof

Let $(M=\textsf{G}/\textsf{L}, Q, \omega , s)$ be a qs-H symmetric space with $\textsf{G}=\textsf{Tr}(M, s)$ and canonical decomposition $\mathfrak {g}=\mathfrak {l}\oplus \mathfrak {m}$, where $\mathfrak {g}={{\,\mathrm{\textsf{Lie}}\,}}(\textsf{G})$ and $\mathfrak {l}={{\,\mathrm{\textsf{Lie}}\,}}(\textsf{L})$ are the corresponding Lie algebras. Let $\sigma (g)=s_{o}\circ g\circ s_o$ be the involution of $\textsf{G}$ defined via the symmetry at the origin $o=e\textsf{L}\in M$. Since $Q\subset {{\,\mathrm{\textsf{End}}\,}}(TM)$ is a $\textsf{G}$-invariant quaternionic structure and ω is a $\textsf{G}$-invariant symplectic 2-form on *M*, the corresponding tiLa is given by $(\mathfrak {g}, \sigma , Q_o, \omega _o)$, where σ is the induced involution on $\mathfrak {g}$ and $(Q_o, \omega _o)$ is the evaluation of the pair (Q,ω) at o∈M. We call $(\mathfrak {g}, \sigma , Q_o, \omega _o)$ the qs-H tiLa associated to $(M=\textsf{G}/\textsf{L}, Q, \omega , s)$ . We will now prove that $(\mathfrak {g}, \sigma , Q_o, \omega _o)$ is unique, up to isomorphism in the category of qs-H tiLas.

**Claim 1:** *Let* (M,Q,ω,s), $(\hat{M}, \hat{Q}, \hat{\omega }, \hat{s})$ *be two simply connected qs-H symmetric spaces and let* $\psi : M\rightarrow \hat{M}$ *be a qs-H isomorphism. Let* o∈M *be a base point in* *M* *with associated qs-H tiLa* $(\mathfrak {g}, \sigma , Q_{o}, \omega _{o})$, *and take as base point in* $\hat{M}$ *the point* $\hat{o}:=\psi (o)$, *with associated qs-H tiLa* $(\hat{\mathfrak {g}}, \hat{\sigma }, \hat{Q}_{\hat{o}}, \hat{\omega }_{\hat{o}})$. *Then the two qs-H tiLas are isomorphic.*

By definition, and by part (3) in Proposition 2.4, the qs-H isomorphism ψ satisfies the relations

$$ \psi \circ s_{x}=\hat{s}_{\psi (x)}\circ \psi , \ \ x\in M\,,\quad \psi ^*\hat{Q}_{\hat{o}}=Q_{o}\,,\quad \psi ^*\hat{\omega }_{\hat{o}}=\omega _{o}\,. $$

Moreover, ψ induces a Lie group isomorphism $\Psi : \textsf{G}\rightarrow \hat{\textsf{G}}$ defined by $\Psi (s_x\circ s_y):=\hat{s}_{\psi (x)}\circ \hat{s}_{\psi (y)}$, for all x,y∈M. Hence Ψ maps the generators of $\textsf{G}={{\,\mathrm{\textsf{Tr}}\,}}(M, s)$ to the generators of $\hat{\textsf{G}}={{\,\mathrm{\textsf{Tr}}\,}}(\hat{M}, \hat{s})$. This satisfies $\Psi \circ \sigma =\hat{\sigma }\circ \Psi$, and it follows that the differential ${{\,\textrm{d}\,}}\Psi _{e} : \mathfrak {g}\rightarrow \hat{\mathfrak {g}}$ is the desired isomorphism between the associated qs-H tiLas $(\mathfrak {g}, \sigma , Q_o, \omega _o)$ and $(\hat{\mathfrak {g}}, \hat{\sigma }, \hat{Q}_{\hat{o}}, \hat{\omega }_{\hat{o}})$. □

This proves the one direction of the statement in the theorem. We will now prove the converse. Let $(\mathfrak {g}, \sigma , Q_{0}, \omega _{0})$ be a qs-H tiLa and let $\mathfrak {g}=\mathfrak {l}\oplus \mathfrak {m}$ be the corresponding $\mathbb {Z}_2$-grading of $\mathfrak {g}$. Let $\textsf{G}$ be the connected and simply connected Lie group corresponding to the Lie algebra $\mathfrak {g}$, and let $\sigma : \textsf{G}\rightarrow \textsf{G}$ be the analytic automorphism of $\textsf{G}$ generated by σ. This is an involutive automorphism of $\textsf{G}$ and since $\textsf{G}$ is simply connected, the fixed point set $\textsf{G}^{\sigma }$ is connected (see [14, p. 293]). Hence, it is clear that $\textsf{L}=\textsf{G}^{\sigma }$ is the analytic subgroup generated by the Lie subalgebra $\mathfrak {l}$ and moreover that the coset space $M:=\textsf{G}/\textsf{L}$ is a simply connected effective symmetric space, see also Theorem 2.2.

Let $o=e\textsf{L}$ be the origin in $M=\textsf{G}/\textsf{L}$. The pair $(Q_{0}, \omega _{0})$ is by definition an $\mathfrak {l}$-invariant linear qs-H structure on $\mathfrak {m}$, which is also $\textsf{L}$-invariant (since $\textsf{L}$ is connected). Moreover, the pair $(Q_{0}, \omega _{0})$ is invariant under the symmetry $s_{o}$. Thus it descends to a $\textsf{G}$-invariant qs-H structure (Q,ω) on $\textsf{G}/\textsf{L}$, with $Q_{o}=Q_{0}$ and $\omega _{o}=\omega _{0}$, respectively, which is invariant under the symmetries. It remains to prove that $(M=\textsf{G}/\textsf{L}, Q, \omega , s)$ is unique up to isomorphism.

**Claim 2:** *Let* $(\mathfrak {g}, \sigma , Q_{0}, \omega _{0})$ *and* $(\hat{\mathfrak {g}}, \hat{\sigma }, \hat{Q}_{0}, \hat{\omega }_{0})$ *be two isomorphic qs-H tiLas. Then the corresponding simply connected qs-H symmetric spaces* $(M=\textsf{G}/\textsf{L}, Q, \omega , s)$ *and* $(\hat{M}=\hat{\textsf{G}}/\hat{\textsf{L}}, \hat{Q}, \hat{\omega }, \hat{s})$ *are isomorphic.*

### Proof

Let us denote the isomorphism between the qs-H tiLas by $\varphi : \mathfrak {g}\rightarrow \hat{\mathfrak {g}}$. By definition, $\varphi \circ \sigma =\hat{\sigma }\circ \varphi$ and $\varphi ^*\hat{Q}_{0}=Q_{0}$, $\varphi ^*\hat{\omega }_{0}=\omega _{0}$. Moreover, φ preserves the $\mathbb {Z}_2$-grading and we have $\varphi (\mathfrak {l})=\hat{\mathfrak {l}}$ and $\varphi (\mathfrak {m})=\hat{\mathfrak {m}}$. Let $\textsf{G}$ and $\hat{\textsf{G}}$ be the connected, simply connected Lie groups with Lie algebras $\mathfrak {g}$ and $\hat{\mathfrak g}$, respectively, and let $\sigma ,\hat{\sigma }$ be the corresponding involutions at the Lie group level. The isomorphism φ integrates to a unique Lie group isomorphism $\Phi : \textsf{G}\rightarrow \hat{\textsf{G}}$, satisfying $\Phi \circ \sigma =\hat{\sigma }\circ \Phi$. Therefore $\Phi (\textsf{G}^\sigma )=\hat{\textsf{G}}^{\hat{\sigma }}$, and in particular $\Phi (\textsf{L})=\hat{\textsf{L}}$. Hence we obtain a diffeomorphism $f : \textsf{G}/\textsf{L}\rightarrow \hat{\textsf{G}}/\hat{\textsf{L}}$ with $f(g\textsf{L})=\Phi (g)\hat{\textsf{L}}$, $g\in \textsf{G}$. Its differential at the origin $o\in \textsf{G}/\textsf{L}$ satisfies ${{\,\textrm{d}\,}}f_{o}=\varphi |_{\mathfrak {m}}$ and since φ is an isomorphism of qs-H tiLas, it follows that $f^*\hat{Q}=Q$ and $f^*\hat{\omega }=\omega$, where (Q,ω) is the $\textsf{G}$-invariant qs-H structure corresponding to $(Q_{0}, \omega _{0})$ and $(\hat{Q}, \hat{\omega })$ is the $\hat{\textsf{G}}$-invariant qs-H structure corresponding to $(\hat{Q}_{0}, \hat{\omega }_{0})$. To complete the proof one can use part (3) of Proposition 2.4, which implies that $f : \textsf{G}/\textsf{L}\rightarrow \hat{\textsf{G}}/\hat{\textsf{L}}$ satisfies the condition $f\circ s_{g\textsf{L}} = \hat{s}_{f(g\textsf{L})}\circ f$ (this also follows from the relation $\Phi \circ \sigma =\hat{\sigma }\circ \Phi$). □

## The classification

In this section we present the complete classification of qs-H symmetric spaces $M=\textsf{G}/\textsf{L}$. For comparison, let us first recall the classification of such symmetric spaces with $\textsf{G}$ semisimple, obtained in [7].

### Theorem 4.1

([7]) A semisimple quaternionic skew-Hermitian symmetric space $(M=\textsf{G}/\textsf{L}, Q, \omega , s)$ is, up to covering, one of the following symmetric spaces:

$$\begin{aligned} M_{1}&={{\,\mathrm{\textsf{SO}}\,}}^*(2n+2)/{{\,\mathrm{\textsf{SO}}\,}}^*(2n)\textsf{U}(1)\,,\\ M_{2}&={{\,\mathrm{\textsf{SU}}\,}}(2+p,q)/({{\,\mathrm{\textsf{SU}}\,}}(2){{\,\mathrm{\textsf{SU}}\,}}(p,q)\textsf{U}(1))\,,\\ M_{3}&={{\,\mathrm{\textsf{SL}}\,}}(n+1,\mathbb {H})/({{\,\mathrm{\textsf{GL}}\,}}(1,\mathbb {H}){{\,\mathrm{\textsf{SL}}\,}}(n,\mathbb {H}))\,. \end{aligned}$$

Moreover, there are the following additional geometric structures provided by the centre of $\textsf{L}$: (see [9])

- $M_1$ admits a distinguished *Q*-compatible invariant complex structure *J* and the corresponding invariant metric $g_{J}=\omega \circ J$ is pseudo-Kähler.
- $M_2$ admits a distinguished invariant complex structure *I* commuting with *Q* and the corresponding invariant metric $g_{I}=\omega \circ I$ is quaternionic pseudo-Kähler. For q=0, the space $M_2$ is compact and $g_{I}$ is quaternionic Kähler.
- $M_3$ admits a distinguished invariant para-complex structure *I* commuting with *Q* and the corresponding invariant metric $g_{I}=\omega \circ I$ is quaternionic pseudo-Kähler of split signature.

The classification of general qs-H symmetric spaces relies on a geometric construction described in [4], which in our case produces local qs-H manifolds (see also [7, Sect. 6.2]). Further, we use the normal forms of general elements in the Lie algebra $\mathfrak {so}^{*}(2n)\oplus \mathfrak {sp}(1)$ (cf. [10]). As we will see below, this construction also yields the semisimple qs-H symmetric spaces described above, hence it provides an alternative proof for this special case.

The starting point is the contact grading of the Lie algebra $\mathfrak {so}^*(2n+4)$, given as follows: We represent $\mathfrak {so}^*(2n+4)$ by quaternionic matrices of the form

$$\begin{aligned} \begin{pmatrix} a &  Y^*\mathbb {j} &  d\\ X &  A &  Y\\ c & -X^*\mathbb {j} &  -a^* \end{pmatrix}, \end{aligned}$$

where $a\in \mathbb {H}$, $X, Y\in \mathbb {H}^n$, $c, d\in \mathbb {R}$ and $A\in \mathfrak {so}^*(2n)$. Here, $\mathbb {j}$ denotes the matrix of the scalar 2-form on $\mathbb {H}^n$ and $\alpha ^*$ denotes the conjugate transpose of α. Then the contact grading is described by

$$ \mathfrak {so}^{*}(2n+4)=\mathfrak {so}^*(2n+4)_{-2}\oplus \mathfrak {so}^{*}(2n+4)_{-1}\oplus \mathfrak {so}^*(2n+4)_{0}\oplus \mathfrak {so}^*(2n+4)_{1}\oplus \mathfrak {so}^*(2n+4)_{2} $$

with

$$\begin{aligned} \mathfrak {so}^{*}(2n+4)_{-2}:= &   \left\{ \begin{pmatrix} 0 &  0 &  0\\ 0 &  0 &  0\\ c &  0 &  0 \end{pmatrix} : c\in \mathbb {R}\right\} \cong \mathbb {R},\\ \mathfrak {so}^{*}(2n+4)_{-1}:= &   \left\{ \begin{pmatrix} 0 &  0 &  0\\ X &  0 &  0\\ 0 &  -X^*\mathbb {j} &  0 \end{pmatrix} : X\in \mathbb {H}^n\right\} \cong \mathbb {H}^n,\\ \mathfrak {so}^{*}(2n+4)_{0}:= &   \left\{ \begin{pmatrix} a &  0 &  0\\ 0 &  A &  0\\ 0 &  0 &  -a^* \end{pmatrix} : a\in \mathbb {H},\; A\in \mathfrak {so}^*(2n)\right\} \cong \mathbb {H}\oplus \mathfrak {so}^*(2n),\\ \mathfrak {so}^{*}(2n+4)_{1}:= &   \left\{ \begin{pmatrix} 0 &  Y^*\mathbb {j} &  0\\ 0 &  0 &  Y\\ 0 &  0 &  0 \end{pmatrix} : Y\in \mathbb {H}^n \right\} \cong \mathbb {H}^n,\\ \mathfrak {so}^{*}(2n+4)_{2}:= &   \left\{ \begin{pmatrix} 0 &  0 &  d\\ 0 &  0 &  0\\ 0 &  0 &  0 \end{pmatrix} : d\in \mathbb {R}\right\} \cong \mathbb {R}\,. \end{aligned}$$

Note that $\mathfrak {g}_{\pm 2}$ are 1-dimensional and the bilinear form (Levi-bracket) $[ \cdot , \cdot ] : \mathfrak {g}_{-1}\times \mathfrak {g}_{-1}\rightarrow \mathfrak {g}_{-2}$ is a $\mathfrak {g}_{-2}$-valued scalar 2-form (in terms of [7]), and hence non-degenerate.

Set $$e:=\begin{pmatrix} 0& 0& 1\\ 0& 0& 0\\ 0& 0& 0 \end{pmatrix}\in \mathfrak {g}_{2}$$ and $$f:= \begin{pmatrix} 0& 0& 0\\ 0& 0& 0\\ 1& 0& 0 \end{pmatrix}\in \mathfrak {g}_{-2}$$. The grading element $h\in \mathfrak {g}_{0}$ is given by

$$ h=[e, f]=\begin{pmatrix} 1&  \quad 0& \quad 0\\ 0& \quad 0& \quad 0\\ 0& \quad 0& \quad -1 \end{pmatrix} $$

which corresponds to a=1 and A=0, that is, $h\in Z(\mathfrak {g}_{0})$. Note also that, $\mathfrak {g}_{-}:=\mathfrak {g}_{-2}\oplus \mathfrak {g}_{-1}$ is a quaternionic Heisenberg algebra, while (*e*, *h*, *f*) is an $\mathfrak {sl}_{2}$-triple, both naturally associated to the contact grading. On the other hand, the subalgebra $\mathfrak {p}:=\mathfrak {g}_{\ge 0}=\mathfrak {g}_{0}\oplus \mathfrak {g}_{1}\oplus \mathfrak {g}_{2}$ is a parabolic subalgebra of $\mathfrak {so}^*(2n+4)$ and we will denote by $P\subset {{\,\mathrm{\textsf{SO}}\,}}^*(2n+4)$ the corresponding parabolic subgroup of ${{\,\mathrm{\textsf{SO}}\,}}^*(2n+4)$. It is well-known that the (4n+1)-dimensional real flag manifold $N:={{\,\mathrm{\textsf{SO}}\,}}^*(2n+4)/P$ admits an invariant contact distribution $\mathscr {D}$ (see [6, Sect. 4.2]),

$$ \mathscr {D}\cong {{\,\mathrm{\textsf{SO}}\,}}^*(2n+4)\times _{P}\big ((\mathfrak {g}_{-1}\oplus \mathfrak {p})/\mathfrak {p}\big )\,. $$

Then, for a sufficiently small open subset U⊂N of *N* and some 1-dimensional subalgebra $\mathfrak {t}\subset \mathfrak {so}^*(2n+4)$ such that the corresponding right-invariant vector fields are transversal to $\mathscr {D}$ everywhere on *U*, there is a local leaf space

$$ M:=\exp (\mathfrak {t})\backslash U $$

such that q:U→M is a line bundle of scalar 2-forms on *M*. Indeed, the quaternionic structure on *M* induced by the isomorphism $({{\,\textrm{d}\,}}q)_{x}(\mathscr {D}_x)\cong T_{q(x)}M$, is independent of the choice of *x* in the fiber of *q*. Moreover, local sections M→U correspond (via the Levi-bracket) to local almost qs-H structures on *M* of torsion type $\mathcal {X}_4$. In particular, there are sections M→U corresponding to local (torsion-free) qs-H structures on *M* described in [4], which form one-parameter families of (local) qs-H structures, where the corresponding scalar 2-forms differ by a positive scalar, see also [8, Remark 1.6.]. Therefore, two subalgebras $\mathfrak {t}$ and $\hat{\mathfrak {t}}$ provide one-parameter families of locally qs-H isomorphic qs-H structures on *M* if and only if they are conjugated by an element of ${{\,\mathrm{\textsf{SO}}\,}}^*(2n+4)$.

We will now show that this construction can provide the full classification of all qs-H tiLas. First we will establish an important bijection.

### Proposition 4.2

There is a bijection between one-parameter families of isomorphism classes of qs-H tiLas $(\mathfrak {g},\sigma ,Q_o, e^{2t}\omega _o)$ $(t\in \mathbb {R})$ and ${{\,\mathrm{\textsf{SO}}\,}}^*(2n+4)$-conjugacy classes of 1-dimensional subalgebras $\mathfrak {t}\subset \mathfrak {so}^*(2n+4)$ represented by elements of the form

4.1 $$\begin{aligned} \tau =\begin{pmatrix} a &  \quad 0& \quad d\\ 0 &  \quad A &  \quad 0\\ 1& \quad 0 &  \quad a \end{pmatrix}, \end{aligned}$$

such that $a\in \mathfrak {sp}(1)$, $A\in \mathfrak {so}^*(2n)$ and $d\in \mathbb {R}$ satisfy the matrix equation

4.2 $$\begin{aligned} Xd+2AXa-Xa^2-A^2X=0 \end{aligned}$$

for all $X\in \mathbb {H}^n$. For such a representative τ, the reductive complement $\mathfrak {m}$ in the canonical decomposition of $\mathfrak {g}$ is given by the above *X*, *a*, *A* as follows:

$$ \mathfrak {m}=\left\{ \begin{pmatrix} 0 &  (AX-Xa)^*\mathbb {j}&  0\\ X &  0 &  AX-Xa\\ 0& -X^*\mathbb {j} &  0 \end{pmatrix} \right\} . $$

### Proof

We start by proving that the one-parameter family of qs-H structures $(Q, e^{2t}\omega )$ $(t\in \mathbb {R})$ on the local leaf space $M=\exp (\mathfrak {t})\backslash U$ of *N* provided by $\mathfrak {t}\subset \mathfrak {so}^*(2n+4)$, with the claimed properties, gives rise to a qs-H symmetric space $(M, Q, e^{2t}\omega , s)$. Recall that the Lie algebra of the qs-H vector fields of $(Q, e^{2t}\omega )$ is isomorphic to the quotient $N_{\mathfrak {so}^*(2n+4)}(\mathfrak {t})/\mathfrak {t}$, see [5]. Consider now elements $\tilde{X}$ in $\mathfrak {so}^*(2n+4)$ of the form

$$\begin{aligned} \begin{pmatrix} 0 &  (AX-Xa)^*\mathbb {j}&  0\\ X &  0 &  AX-Xa\\ 0& -X^*\mathbb {j} &  0 \end{pmatrix}\,, \end{aligned}$$

where $X\in \mathbb {H}^n$ and *A*, *a* are given by the claimed representative τ of $\mathfrak {t}$, see (4.1). Then, under our assumptions we get that

$$ {[}\tilde{X}, \tau ]=\begin{pmatrix} 0 &  (Xd+2AXa-Xa^2-A^2X)^*\mathbb {j} &  0\\ 0 &  0 &  Xd+2AXa-Xa^2-A^2X\\ 0&  0 &  0 \end{pmatrix}=0\,. $$

Therefore $\tilde{X}\in Z_{\mathfrak {so}^*(2n+4)}(\mathfrak {t})/\mathfrak {t}\subset N_{\mathfrak {so}^*(2n+4)}(\mathfrak {t})/\mathfrak {t}$ for all $X\in \mathbb {H}^n$. Denote by

$$ \mathfrak {m}\subset Z_{\mathfrak {so}^*(2n+4)}(\mathfrak {t})/\mathfrak {t} $$

the space spanned by all such $\tilde{X}$. Then $\mathfrak {g}=\mathfrak {m}\oplus [\mathfrak {m},\mathfrak {m}]$ is a tiLa with involution defined as $-{{\,\mathrm{\textsf{id}}\,}}_{\mathfrak {m}}$ in the $\mathfrak {m}$-part and by ${{\,\mathrm{\textsf{id}}\,}}_{ [\mathfrak {m},\mathfrak {m}]}$ in the $[\mathfrak {m}, \mathfrak {m}]$-part. This is because of the inclusion $[[\mathfrak {m},\mathfrak {m}],\mathfrak {m}]\subset \mathfrak {m}$, which follows as there is no element of $\mathfrak {so}^*(2n+4)_{1}$ in $Z_{\mathfrak {so}^*(2n+4)}(\mathfrak {t})/\mathfrak {t}.$ Then, by construction, this is a qs-H tiLa. Indeed, we have $\omega _o\in \Lambda ^2 \mathfrak {m}^*\otimes \mathfrak {t}$, which corresponds to the Levi-bracket and is preserved by the symmetries (because $[\mathfrak {m},\mathfrak {m}]\subset Z_{\mathfrak {so}^*(2n+4)}(\mathfrak {t})/\mathfrak {t}$).

Let us now consider two abelian subalgebras $\mathfrak {t},\hat{\mathfrak {t}}\subset \mathfrak {so}^*(2n+4)$, represented by *a*, *A*, *d* and $\hat{a},\hat{A},\hat{d}$, respectively. Suppose that they are conjugated by an element $g\in {{\,\mathrm{\textsf{SO}}\,}}^*(2n+4)$. In fact, this element has to belong in ${{\,\mathrm{\textsf{SL}}\,}}(2,\mathbb {R})\times {{\,\mathrm{\textsf{Sp}}\,}}(1){{\,\mathrm{\textsf{SO}}\,}}^*(2n)$, where ${{\,\mathrm{\textsf{SL}}\,}}(2,\mathbb {R})$ corresponds to the $\mathfrak {sl}_{2}$-triple (*e*, *h*, *f*). This is because otherwise the representatives given by *a*, *A*, *d* and $\hat{a},\hat{A},\hat{d}$ would not be mapped onto each other. Note that the action of ${{\,\mathrm{\textsf{SL}}\,}}(2,\mathbb {R})$ maps *a*, *A*, *d* (after rescaling) to $e^{2t}a, e^{2t}A, e^{4t}d$, for some $t\in \mathbb {R}$. This induces a Lie algebra isomorphism $t{{\,\mathrm{\textsf{id}}\,}}_{\mathfrak {m}}\oplus {{\,\mathrm{\textsf{id}}\,}}_{\mathfrak {l}}$ between the corresponding tiLas which rescales the scalar 2-form, i.e., preserves the one-parameter family. On the other hand, the action of $(b,B)\in {{\,\mathrm{\textsf{Sp}}\,}}(1){{\,\mathrm{\textsf{SO}}\,}}^*(2n)$ maps *a*, *A*, *d* to $bab^{-1},BAB^{-1},d$, and this induces an isomorphism of qs-H tiLas via the adjoint action of ${{\,\mathrm{\textsf{Sp}}\,}}(1){{\,\mathrm{\textsf{SO}}\,}}^*(2n)$. Thus we conclude that

4.3 $$\begin{aligned} \hat{a}=e^{2t}bab^{-1}\,,\quad \hat{A}=e^{2t}BAB^{-1}\,,\quad \hat{d}=e^{4t}d \end{aligned}$$

and the corresponding qs-H tiLas belong to the same one-parameter family.

Conversely, for any almost qs-H manifold (M,Q,ω) of dimension 4*n* there is a construction given in [5, Theorem 4] that provides a parabolic contact structure of type $({{\,\mathrm{\textsf{SO}}\,}}^*(2n+4), P)$, which contains a line bundle q:U→M over *M* and a contact distribution $\mathcal {D}$ on total space *U*. This construction depends only on the conformal class of ω, hence the result will be the same for the whole one-parameter family $e^{2t}\omega$ of scalar 2-forms. Moreover, by [5, Theorem 5] it follows that every qs-H transformation lifts to an isomorphism of the parabolic contact structures. In particular, for a qs-H symmetric space $(M,Q, e^{2t}\omega , s)$ in the one-parameter family and every y∈U, there exists a unique automorphism $s_y$ of the parabolic contact structure such that $s_y(y)=y$ and $s_y$ is a lift of $s_{q(y)}$, i.e.,

4.4 $$\begin{aligned} ({{\,\textrm{d}\,}}s_y)_{y}|_{\mathcal {D}_{y}}=-{{\,\mathrm{\textsf{id}}\,}}_{\mathcal {D}_y}\,. \end{aligned}$$

At this point, recall that the fundamental invariant of the parabolic contact structure is the harmonic torsion (in terms of [6, p. 419]) which is a special section of the vector bundle $\Lambda ^2 \mathcal {D}^*\otimes \mathcal {D}$. Since by (4.4) the differential $({{\,\textrm{d}\,}}s_y)_{y}|_{\mathcal {D}_{y}}$ acts as $-{{\,\mathrm{\textsf{id}}\,}}$ on $\Lambda ^2 \mathcal {D}^*\otimes \mathcal {D}$, this harmonic torsion should vanish. Therefore, the parabolic contact structure is locally isomorphic to the homogeneous space $N={{\,\mathrm{\textsf{SO}}\,}}^*(2n+4)/P$, that is, $\mathcal {D}\cong \mathscr {D}$. Then, by construction [5, Theorem 4], there is an abelian subalgebra $\mathfrak {t}\subset \mathfrak {so}^*(2n+4)$ such that the corresponding right-invariant vector fields are vertical, i.e., belong to ${{\,\mathrm{\textsf{Ker}}\,}}({{\,\textrm{d}\,}}q)$. Moreover, the qs-H tiLa $(\mathfrak {g},\sigma ,Q_o, e^{2t}\omega _o)$ corresponding to the qs-H symmetric space $(M,Q, e^{2t}\omega ,s)$ lifts to a subalgebra $\mathfrak {g}'\subset N_{\mathfrak {so}^*(2n+4)}(\mathfrak {t})$. Then σ corresponds to the involution induced by conjugation by the element $-{{\,\mathrm{\textsf{id}}\,}}\in {{\,\mathrm{\textsf{SO}}\,}}^*(2n)\subset P.$ Therefore, after conjugation by an element of the subgroup $\exp (\mathfrak {so}^*(2n+4)_2)\subset P$, we can bring $\mathfrak {t}$ into a form represented by *a*, *A*, *d* as in (4.1). Moreover, there is a lift $\mathfrak {m}' \subset N_{\mathfrak {so}^*(2n+4)}(\mathfrak {t})$ of the reductive complement $\mathfrak {m}$ that takes the form

$$\begin{aligned} \begin{pmatrix} 0 &  \quad f(X)^*\mathbb {j}&  \quad 0\\ X &  \quad 0 &  \quad f(X)\\ 0& \quad -X^*\mathbb {j} &  \quad 0 \end{pmatrix}\,, \end{aligned}$$

for all $X\in \mathbb {H}^n$ and some linear map $f : \mathbb {H}^n\rightarrow \mathbb {H}^n$. This is because the parts $\mathfrak {so}^*(2n+4)_{\pm 1}$ are the (-1)-eigenspaces of the corresponding involution. Then, a direct computation shows that the condition $[\mathfrak {m}',\mathfrak {t}]=0$ implies that f(X)=AX-Xa and $Xd+2AXa-Xa^2-A^2X=0$, for all $X\in \mathbb {H}^n.$

Recall now that by Theorem 3.4 an isomorphism of qs-H tiLas corresponds to a qs-H isomorphism between the corresponding symmetric spaces. Moreover, by [5, Theorem 5] and [6, Proposition 1.5.2] this isomorphism should lift to left multiplication by an element of ${{\,\mathrm{\textsf{SO}}\,}}^*(2n+4)$. This shows that the corresponding abelian subalgebra $\mathfrak {t}$ should belong to the same ${{\,\mathrm{\textsf{SO}}\,}}^*(2n+4)$-conjugacy class and completes the proof. □

Let us now use Proposition 4.2 to construct examples of qs-H tiLas. As we will see later, these examples exhaust all possibilities and yield the full classification of qs-H tiLas. Next we will denote by $I_{p,q}$ the diagonal matrix with *p* ones and *q* negative ones on the diagonal.

### Example 4.3

Let *n* be even and set $$\mathbb {j}=\begin{pmatrix} 0 &  I_{\frac{n}{2},0} \\ I_{0,\frac{n}{2}} &  0\\ \end{pmatrix}$$, that is, we identify $\mathbb {H}^n$ with the linear model $[{{\,\mathrm{\textsf{E}}\,}}{{\,\mathrm{\textsf{H}}\,}}]$ using a quaternionic Darboux basis, see [7, Sect. A.2]. Let also *p*, *q* be integer parameters such that $0\le p,q,p+q \le \frac{n}{2}$ and consider the generators τ of $\mathfrak {t}$ of the form

$$\begin{aligned} \tau =\begin{pmatrix} 0& \quad 0&  \quad 0&  \quad 0&  \quad 0& \quad 0& \quad 0& \quad 0\\ 0&  \quad 0&  \quad 0& \quad 0& \quad 0&  \quad 0& \quad 0&  \quad 0\\ 0& \quad 0& \quad 0& \quad 0&  0& \quad I_{q,0}& \quad 0& \quad 0\\ 0& \quad 0&  \quad 0& \quad 0&  0& \quad 0& \quad 0& \quad 0\\ 0&  \quad I_{p,0}&  0& \quad 0& \quad 0& \quad 0& \quad 0& \quad 0\\ 0&  \quad 0&  \quad 0& \quad 0& \quad 0& \quad 0& \quad 0& \quad 0\\ 0&  \quad 0&  \quad 0& \quad 0&  \quad 0& \quad 0& \quad 0& \quad 0\\ 1&  \quad 0&  \quad 0& \quad 0&  \quad 0& \quad 0& \quad 0& \quad 0 \end{pmatrix}\,, \end{aligned}$$

that is, τ admits a block decomposition with blocks of sizes $1,p,q,\frac{n}{2}-(p+q),p,q,\frac{n}{2}-(p+q),1$ respectively. Hence τ is a (n+2)×(n+2) quaternionic matrix. It is easy to see that τ satisfies (4.2), hence by Proposition 4.2 we obtain a qs-H tiLa where the Lie algebra $\mathfrak {g}$ is defined by $\mathfrak {g}=\big ([\mathfrak {m}, \mathfrak {m}]/\mathfrak {t}\big )\oplus \mathfrak {m}$, where $\mathfrak {m}$ has the block matrix presentation

$$ \mathfrak {m}=\begin{pmatrix} 0 &  -X_1^*&  0& 0&  0& X_2^*& 0& 0\\ X_1&  0 &  0& 0&  0&  0 & 0& 0\\ Y_1&  0 &  0 &  0&  0 &  0 &  0 & X_2\\ Z_1&  0 &  0& 0&  0&  0& 0& 0\\ Y_2&  0 &  0 &  0 &  0 &  0 &  0 & X_1\\ X_2& 0 &  0& 0 & 0&  0& 0& 0\\ Z_2&  0&  0& 0&  0& 0& 0& 0\\ 0 &  Y_2^*&  X_2^*& Z_2^* & -X_1^*& -Y_1^*& -Z_1^*& 0 \end{pmatrix}. $$

Note that the blocks of $\mathfrak {m}$ have the same size with the blocks of the representative τ. It follows that $\mathfrak {g}$ admits the block matrix presentation

$$ \mathfrak {g}=\begin{pmatrix} S_1&  -X_1^*&  0& 0&  0& X_2^*& 0& 0\\ X_1&  S_2&  0& 0&  0& S_3^*& 0& 0\\ Y_1&  H_1&  S_4&  R_1 &  S_3 &  H_2 &  R_2^*& X_2\\ Z_1&  R_3&  0& 0&  0&  R_{2}& 0& 0\\ Y_2&  H_3&  S_3^*&  R_4^* &  S_2&  -H_1^*& -R_3^*& X_1\\ X_2&  S_3&  0& 0 & 0&  S_4& 0& 0\\ Z_2&  R_4&  0& 0&  0& -R_1^*& 0& 0\\ H_4 &  Y_2^*&  X_2^*& Z_2^* & -X_1^*& -Y_1^*& -Z_1^*& S_1 \end{pmatrix}/\mathfrak {t} \,. $$

Here, we have $X_a, Y_a, Z_a\in \mathfrak {m}$ with $1\le a\le 2$ and $S_b, H_b, R_b\in [\mathfrak {m}, \mathfrak {m}]$, respectively, for $1\le b\le 4$, with

$$ S_1=-S_1^*,\quad S_2=-S_2^*, \quad S_4=-S_4^*,\quad H_2=H_2^*,\quad H_3=H_3^*, \quad H_4=H_4^*\,. $$

Then we see that the matrices $X_1, X_2, S_1, \ldots , S_4$ span the semisimple part of $\mathfrak {g}$, which is isomorphic to $\mathfrak {sp}(p+1, q)$. Moreover, the radical $\mathfrak {r}\subset \mathfrak {g}$ of $\mathfrak {g}$ decomposes into two distinguished parts

$$ \mathfrak {r}=\mathfrak {r}_\text {qH}/\mathfrak {t}\oplus \mathfrak {r}_\text {deg}\,, $$

where $\mathfrak {r}_\text {qH}$ is generated by the matrices $Y_1, Y_2, H_1, \ldots , H_4$ and $\mathfrak {r}_\text {deg}$ is generated by $Z_1, Z_2, R_1, \ldots , R_4$. Note that $\mathfrak {r}_\text {qH}$ can be arranged into a block matrix decomposition of sizes 1, *p*, *q*, i.e.,

$$ \begin{pmatrix} H_4 &  Y_2^* &  -Y_1^*\\ Y_1&  H_1 &  H_2\\ Y_2&  H_3 &  -H_1^* \end{pmatrix}. $$

By dimensional reasons, it turns out that this is isomorphic to the space of quaternionic Hermitian scalar products on the standard representation of $\mathfrak {sp}(p+1, q)$. In particular, τ belongs to $\mathfrak {r}_\text {qH}$ and represents the non-degenerate quaternionic Hermitian scalar product defining the Lie algebra $\mathfrak {sp}(p+1, q)$. We mention that:

∙ For p+q=0, we have $\tau =H_4$ and the Lie algebra $\mathfrak {g}$ is exactly the linear model $[{{\,\mathrm{\textsf{E}}\,}}{{\,\mathrm{\textsf{H}}\,}}]$.

∙ For general *p*, *q* the orbit of the semisimple part $\mathfrak {sp}(p+1, q)$ is the quaternionic pseudo-Kähler symmetric space ${{\,\mathrm{\textsf{Sp}}\,}}(p+1, q)/({{\,\mathrm{\textsf{Sp}}\,}}(1){{\,\mathrm{\textsf{Sp}}\,}}(p, q))$.

Let us now highlight a few low-dimensional cases, where special isomorphisms of simple Lie algebras occur.

1. Let us consider the case n=2, p=1 and q=0. In this case, the block sizes are (1, 1, 0, 0, 1, 0, 0, 1) and Lie algebra $\mathfrak {g}$ has the matrix presentation
  $$ \mathfrak {g}=\left\{ \begin{pmatrix} S_1 &  -X_1^* &  0 &  0\\ X_1 &  S_2 &  0 &  0\\ Y_2 &  H_3 &  S_2 &  X_1\\ H_4 &  Y_2^* &  -X_1^* &  S_1 \end{pmatrix} : S_1, S_2\in \mathfrak {sp}(1), X_1, Y_2\in \mathbb {H}, H_3, H_4\in \mathbb {R}\right\} /\mathfrak {t}\,, $$
  thus $\mathfrak {g}$ is 15-dimensional. Using the isomorphism $\mathfrak {sp}(2)\cong \mathfrak {so}(5)$ we may express the Levi decomposition as $\mathfrak {g}=\mathfrak {so}(5)\oplus \mathbb {R}^5$, where $\mathfrak {so}(5)$ acts on $\mathbb {R}^{5}$ by the standard representation. Moreover, we see that $\mathfrak {l}=\mathfrak {so}(4)\oplus \mathbb {R}$, and the reductive complement $\mathfrak {m}$ is 8-dimensional,
  $$ \mathfrak {m}=\left\{ \begin{pmatrix} 0 &  -X_1^* &  0 &  0\\ X_1 &  0 &  0 &  0\\ Y_2 &  0 &  0 &  X_1\\ 0 &  Y_2^* &  -X_1^* &  0 \end{pmatrix} : X_1, Y_2\in \mathbb {H}\right\} \,. $$
  This yields the non-semisimple symmetric space
  $$ ({{\,\mathrm{\textsf{SO}}\,}}(5)\ltimes \mathbb {R}^5)/({{\,\mathrm{\textsf{SO}}\,}}(4)\times \mathbb {R})\,. $$
  In this case, the semisimple quaternionic Kähler symmetric space is the coset ${{\,\mathrm{\textsf{SO}}\,}}(5)/{{\,\mathrm{\textsf{SO}}\,}}(4)$, that is, the 4-sphere $\textsf{S}^4\cong \mathbb {H}\textsf{P}^1$.
2. Let us now consider the case n=2, p=0 and q=1. Here the sizes of the blocks are (1, 0, 1, 0, 0, 1, 0, 1) and Lie algebra $\mathfrak {g}$ has the matrix presentation
  $$ \mathfrak {g}=\left\{ \begin{pmatrix} S_1&  0 &  X_2^*&  0\\ Y_1&  S_4&  H_2 & X_2\\ X_2&  0&  S_4&  0 \\ H_4 &  X_2^*&  -Y_1^* & S_1 \end{pmatrix} : S_1, S_4\in \mathfrak {sp}(1), X_2, Y_1\in \mathbb {H}, H_2, H_4\in \mathbb {R}\right\} /\mathfrak {t} \,. $$
  Hence $\mathfrak {g}$ is again 15-dimensional. This time, using the isomorphism $\mathfrak {sp}(1, 1)\cong \mathfrak {so}(4, 1)$ we can express the Levi decomposition of $\mathfrak {g}$ as $\mathfrak {g}=\mathfrak {so}(4, 1)\oplus \mathbb {R}^5$, where $\mathbb {R}^5$ is the standard representation of $\mathfrak {so}(4, 1)$. In this case we get $\mathfrak {l}=\mathfrak {so}(4)\oplus \mathbb {R}$ and
  $$ \mathfrak {m}=\left\{ \begin{pmatrix} 0&  0 &  X_2^*&  0\\ Y_1&  0&  0 & X_2\\ X_2&  0&  0&  0 \\ 0 &  X_2^*&  -Y_1^* &  0 \end{pmatrix} : X_2, Y_1\in \mathbb {H}\right\} \,. $$
  This corresponds to the 8-dimensional non-semisimple symmetric space
  $$ ({{\,\mathrm{\textsf{SO}}\,}}(4, 1)\ltimes \mathbb {R}^5)/({{\,\mathrm{\textsf{SO}}\,}}(4)\times \mathbb {R}) $$
  where the semisimple quaternionic Kähler symmetric space is the non-compact dual of the previous one, that is, the symmetric space ${{\,\mathrm{\textsf{SO}}\,}}(4, 1)/{{\,\mathrm{\textsf{SO}}\,}}(4)$, which up to covering is the 1-dimensional quaternionic hyperbolic space ${{\,\mathrm{\textsf{Sp}}\,}}(1, 1)/({{\,\mathrm{\textsf{Sp}}\,}}(1)\times {{\,\mathrm{\textsf{Sp}}\,}}(1))$.

### Example 4.4

Suppose that *n* is odd and set

$$ \mathbb {j}=\begin{pmatrix} 0 &  \quad 0& \quad I_{\frac{n-1}{2},0} \\ 0& \quad j&  \quad 0\\ I_{0,\frac{n-1}{2}} & \quad 0&  \quad 0 \end{pmatrix}\,. $$

This choice identifies $\mathbb {H}^n$ with the linear model $[{{\,\mathrm{\textsf{E}}\,}}{{\,\mathrm{\textsf{H}}\,}}]$ by a generalization of a quaternionic Darboux basis, see [7, Proposition A.9]. Let *p*, *q* be integer parameters such that $0\le p,q,p+q \le \frac{n-1}{2}$, and consider the generator $\tau \in \mathfrak {t}$ of the form

$$\begin{aligned} \tau =\begin{pmatrix} 0&  0&  0& 0&  0& 0& 0& 0& 0\\ 0&  0&  0& 0&  0& 0& 0& 0& 0\\ 0&  0&  0& 0&  0& 0& I_{q,0}& 0& 0\\ 0&  0&  0& 0&  0& 0& 0& 0& 0\\ 0&  0&  0& 0&  0& 0& 0& 0& 0\\ 0&  I_{p,0}&  0& 0&  0& 0& 0& 0& 0\\ 0&  0&  0& 0&  0& 0& 0& 0& 0\\ 0&  0&  0& 0&  0& 0& 0& 0& 0\\ 1&  0&  0& 0&  0& 0& 0& 0& 0 \end{pmatrix}. \end{aligned}$$

Hence, in this case the (n+2)×(n+2) quaternionic matrix τ admits a block decomposition with blocks of sizes $1,p,q,\frac{n-1}{2}-(p+q),1,p,q,\frac{n-1}{2}-(p+q),1$. It is easy to see that τ satisfies (4.2), hence by Proposition 4.2 we obtain a qs-H tiLa with Lie algebra $\mathfrak {g}$ defined by $\mathfrak {g}=\big ([\mathfrak {m}, \mathfrak {m}]/\mathfrak {t}\big )\oplus \mathfrak {m}$, where the reductive complement $\mathfrak {m}$ is given by

$$ \mathfrak {m}=\begin{pmatrix} 0&  -X_1^*&  0& 0&  0& 0& X_2^*& 0& 0\\ X_1& 0&  0& 0&  0& 0&  0& 0& 0\\ Y_1&  0&  0&  0 &  0&  0 &  0 &  0& X_2\\ Z_1&  0&  0& 0&  0&  0&  0& 0& 0\\ Z_3&  0&  0&  0&  0& 0 &  0&  0&  0\\ Y_2&  0&  0&  0 & 0 &  0& 0& 0& X_1\\ X_2& 0&  0& 0 & 0 &  0&  0& 0& 0\\ Z_2&  0&  0& 0&  0&  0&  0& 0& 0\\ 0 &  Y_2^*&  X_2^*&  Z_2^* &  -Z_3^*j &  -X_1^*& -Y_1^*& -Z_1^*& 0 \end{pmatrix}. $$

Note that the block structure of $\mathfrak {m}$ matches the block sizes of the representative τ. It follows that $\mathfrak {g}$ admits the following block matrix presentation

$$ \mathfrak {g}=\begin{pmatrix} S_1&  -X_1^*&  0& 0&  0& 0& X_2^*& 0& 0\\ X_1&  S_2&  0& 0&  0& 0&  S_3^*& 0& 0\\ Y_1&  H_1&  S_4&  R_1 &  R_6&  S_3 &  H_2 &  R_2^*& X_2\\ Z_1&  R_3&  0& 0&  0&  0&  R_{2}& 0& 0\\ Z_3&  R_5&  0&  0&  0& 0 &  jR_6^*&  0&  0\\ Y_2&  H_3&  S_3^*&  R_4^* & -R_5^*j &  S_2&  -H_1^*& -R_3^*& X_1\\ X_2&  S_3&  0& 0 & 0 &  0&  S_4& 0& 0\\ Z_2&  R_4&  0& 0&  0&  0&  -R_1^*& 0& 0\\ H_4 &  Y_2^*&  X_2^*&  Z_2^* &  -Z_3^*j &  -X_1^*& -Y_1^*& -Z_1^*& S_1 \end{pmatrix}/\mathfrak {t} $$

with $X_1, X_2, Y_1, Y_2, Z_1, \ldots , Z_3\in \mathfrak {m}$ and $S_b, H_b, R_1,\ldots , R_6\in [\mathfrak {m}, \mathfrak {m}]$, respectively, for $1\le b\le 4$, with

$$ S_1=-S_1^*,\quad S_2=-S_2^*, \quad S_4=-S_4^*,\quad H_2=H_2^*,\quad H_3=H_3^*, \quad H_4=H_4^*\,. $$

Similarly with the previous example we see that that the matrices $X_1, X_2, S_1, \ldots , S_4$ span the semisimple part of $\mathfrak {g}$, which is isomorphic to $\mathfrak {sp}(p+1, q)$. Moreover, in this case the decomposition $\mathfrak {r}=\mathfrak {r}_\text {qH}/\mathfrak {t}\oplus \mathfrak {r}_\text {deg}$ of the radical $\mathfrak {r}\subset \mathfrak {g}$ of $\mathfrak {g}$ is such that $\mathfrak {r}_\text {qH}=\text {span}\{Y_1, Y_2, H_1, \ldots , H_4\}$ and $\mathfrak {r}_\text {deg}=\text {span}\{Z_1, Z_2, Z_3, R_1, \ldots , R_6\}$, respectively. Again the component $\mathfrak {r}_\text {qH}$ is isomorphic to the space of quaternionic Hermitian scalar products on the standard representation of $\mathfrak {sp}(p+1, q)$, where $\tau \in \mathfrak {r}_\text {qH}$ represents the non-degenerate quaternionic Hermitian scalar product defining the Lie algebra $\mathfrak {sp}(p+1, q)$. In particular, for p+q=0, we similarly obtain $\tau =H_4$ and the Lie algebra $\mathfrak {g}$ is exactly the linear model $[{{\,\mathrm{\textsf{E}}\,}}{{\,\mathrm{\textsf{H}}\,}}]$, while for general *p*, *q* the orbit of the semisimple part $\mathfrak {sp}(p+1, q)$ is once more the quaternionic pseudo-Kähler symmetric space ${{\,\mathrm{\textsf{Sp}}\,}}(p+1, q)/({{\,\mathrm{\textsf{Sp}}\,}}(1){{\,\mathrm{\textsf{Sp}}\,}}(p, q))$.

Let us discuss a low-dimensional case. Suppose that n=3, p=1, q=0. In this case $\mathfrak {g}$ has the matrix presentation

$$ \mathfrak {g}=\left\{ \begin{pmatrix} S_1&  -X_1^* &  0& 0 & 0\\ X_1&  S_2 &  0& 0 & 0\\ Z_3&  R_5 &  0& 0 &  0\\ Y_2&  H_3 & -R_5^*j &  S_2 & X_1\\ -H_3 &  Y_2^* &  -Z_3^*j &  -X_1^*&  S_1 \end{pmatrix} : X_1, Y_2, Z_3, R_5\in \mathbb {H}, S_1, S_2\in \mathfrak {sp}(1), H_3\in \mathbb {R}\right\} $$

and we obtain a 12-dimensional non-semisimple qs-H symmetric space with reductive complement $\mathfrak {m}$ having the form

$$ \mathfrak {m}=\left\{ \begin{pmatrix} 0&  -X_1^* &  0& 0 & 0\\ X_1& 0 &  0& 0 & 0\\ Z_3&  0 &  0& 0 &  0\\ Y_2&  0 &  0 &  0 & X_1\\ 0 &  Y_2^* &  -Z_3^*j &  -X_1^*& 0 \end{pmatrix} : X_1, Y_2, Z_3\in \mathbb {H}\right\} \,. $$

Note that the block $$\begin{pmatrix} S_1 &  -X_1^*\\ X_1 &  S_2\end{pmatrix}$$ represents the semisimple part, which corresponds to the quaternionic Kähler symmetric space $\mathbb {H}\textsf{P}^{1}={{\,\mathrm{\textsf{Sp}}\,}}(2)/({{\,\mathrm{\textsf{Sp}}\,}}(1)\times {{\,\mathrm{\textsf{Sp}}\,}}(1))$. The solvable part of $\mathfrak {g}$, as a vector space, is the direct sum of the standard representation $\mathbb {H}^2$ of ${{\,\mathrm{\textsf{Sp}}\,}}(2)$ and the space of the quaternionic Hermitian scalar products on $\mathbb {H}^2$. However, observe that the Lie bracket on the solvable part contains the pairing

$$ {[}(u, v), (w, z)]=\begin{pmatrix} z^*ju-v^*jw &  z^*jv-w^*jz\\ w^*ju-u^*jw &  w^*jv-u^*jz \end{pmatrix}/(\mu {{\,\mathrm{\textsf{Id}}\,}})\,,\quad \text {for}\quad (u, v), (w, z)\in \mathbb {H}^2, \mu \in \mathbb {R}\,, $$

where $\mu {{\,\mathrm{\textsf{Id}}\,}}$ represents $\mathfrak {t}$, hence in this case the radical is not abelian.

Let us now describe the realization of the semisimple qs-H tiLas (see Theorem 4.1) via the approach that described in Proposition 4.2.

### Example 4.5

For general n>1 consider the (n+2)×(n+2) quaternionic matrix

$$ \tau =\begin{pmatrix} i &  \quad 0& \quad -1\\ 0 &  \quad 0 &  \quad 0\\ 1& \quad 0 & \quad i \end{pmatrix} $$

with a block decomposition into blocks of sizes 1, *n*, 1. Setting $\mathbb {j}=j$ we can identify $\mathbb {H}^n$ with the linear model $[{{\,\mathrm{\textsf{E}}\,}}{{\,\mathrm{\textsf{H}}\,}}]$ by means of a skew-Hermitian basis, see [7, Sect. A.2]. It is easy to see that τ satisfies (4.2), hence by Proposition 4.2 we obtain a qs-H tiLa with Lie algebra $\mathfrak {g}$ defined by $\mathfrak {g}=\big ([\mathfrak {m}, \mathfrak {m}]/\mathfrak {t}\big )\oplus \mathfrak {m}$, where this time the reductive complement $\mathfrak {m}$ is given by

$$ \mathfrak {m}=\left\{ \begin{pmatrix} 0 &  iX^*j &  0 \\ X &  0 &  -Xi \\ 0 &  -X^*j &  0 \end{pmatrix} : X\in \mathbb {H}^n\right\} . $$

Again the block structure of $\mathfrak {m}$ matches the block sizes of τ. It follows that $\mathfrak {g}$ admits the following block matrix presentation

$$ \mathfrak {g}=\left\{ \begin{pmatrix} S_1 &  iX^*j &  0 \\ X &  S_2 &  -Xi \\ 0 &  -X^*j &  S_1 \end{pmatrix} : S_1\in \mathfrak {u}(1), S_2\in \mathfrak {so}^*(2n), X\in \mathbb {H}^n\right\} , $$

which, obviously, corresponds to the simple qs-H symmetric space ${{\,\mathrm{\textsf{SO}}\,}}^*(2n+2)/{{\,\mathrm{\textsf{SO}}\,}}^*(2n)\textsf{U}(1).$

### Example 4.6

For n=p+q>1 consider the (n+2)×(n+2) quaternionic matrix

$$ \tau =\begin{pmatrix} 0 &  \quad 0& \quad -1\\ 0 &  \quad jI_{p,q} &  \quad 0\\ 1& \quad 0 &  \quad 0 \end{pmatrix} $$

with a block decomposition into blocks of sizes 1, *n*, 1. Moreover, set $\mathbb {j}=j$ such that we can identify $\mathbb {H}^n$ with $[{{\,\mathrm{\textsf{E}}\,}}{{\,\mathrm{\textsf{H}}\,}}]$ by means of a skew-Hermitian basis. The matrix τ satisfies (4.2), hence once more by Proposition 4.2 we obtain a qs-H tiLa with Lie algebra $\mathfrak {g}$ defined by $\mathfrak {g}=\big ([\mathfrak {m}, \mathfrak {m}]/\mathfrak {t}\big )\oplus \mathfrak {m}$. In this case the reductive complement $\mathfrak {m}$ is given by

$$ \mathfrak {m}=\left\{ \begin{pmatrix} 0 &  X^*I_{p, q} &  0 \\ X &  0 &  jI_{p, q}X \\ 0 &  -X^*j &  0 \end{pmatrix} : X\in \mathbb {H}^n\right\} , $$

where the blocks have the same size as the blocks of τ. It follows that $\mathfrak {g}$ admits the following block matrix presentation

$$ \mathfrak {g}=\left\{ \begin{pmatrix} S_1 &  X^*I_{p, q} &  0 \\ X &  S_2 &  jI_{p, q}X \\ 0 &  -X^*j &  S_1 \end{pmatrix} : S_1\in \mathfrak {sp}(1), S_2\in \mathfrak {u}(p, q), X\in \mathbb {H}^n\right\} \,. $$

This corresponds to the simple qs-H symmetric space ${{\,\mathrm{\textsf{SU}}\,}}(2n+2)/({{\,\mathrm{\textsf{SU}}\,}}(2){{\,\mathrm{\textsf{SU}}\,}}(p,q)\textsf{U}(1)).$

### Example 4.7

For any even *n* consider the (n+2)×(n+2) quaternionic matrix τ

$$ \tau =\begin{pmatrix} 0 & 0& 0&  1\\ 0 &  I_{\frac{n}{2},0} & 0&  0\\ 0 & 0&  I_{0,\frac{n}{2}} &  0\\ 1& 0 &  0 &  0 \end{pmatrix} $$

with block decomposition of sizes $1,\frac{n}{2},\frac{n}{2},1$. Moreover, identify $\mathbb {H}^n$ with $[{{\,\mathrm{\textsf{E}}\,}}{{\,\mathrm{\textsf{H}}\,}}]$ by means of a quaternionic Darboux basis, by setting

$$ \mathbb {j}=\begin{pmatrix} 0 &  I_{\frac{n}{2},0} \\ I_{0,\frac{n}{2}} &  0\\ \end{pmatrix}. $$

Again, the matrix τ satisfies (4.2) and Proposition 4.2 gives a qs-H tiLa with Lie algebra $\mathfrak {g}$ defined by $\mathfrak {g}=\big ([\mathfrak {m}, \mathfrak {m}]/\mathfrak {t}\big )\oplus \mathfrak {m}$. In this case the reductive complement $\mathfrak {m}$ is given by

$$ \mathfrak {m}=\left\{ \begin{pmatrix} 0 &  X_2^* &  X_1^* &  0 \\ X_1 &  0 &  0 &  X_1 \\ X_2 &  0 &  0 &  -X_2 \\ 0 &  X_2^* &  -X_1^* &  0 \end{pmatrix} : X_1, X_2\in \mathbb {H}^{\frac{n}{2}}\right\} , $$

where the blocks have the same size as the blocks of τ. Therefore, for the Lie algebra $\mathfrak {g}$ we obtain the block matrix presentation

$$ \mathfrak {g}=\left\{ \begin{pmatrix} S_1 &  X_2^* &  X_1^* &  0 \\ X_1 &  S_2 &  0 &  X_1 \\ X_2 &  0 &  -S_2^* &  -X_2 \\ 0 &  X_2^* &  -X_1^* &  S_1 \end{pmatrix} : S_1\in \mathfrak {sp}(1), S_2\in \mathfrak {gl}(\frac{n}{2}, \mathbb {H}), X_1, X_2\in \mathbb {H}^{\frac{n}{2}}\right\} \,. $$

This corresponds to the simple symmetric space ${{\,\mathrm{\textsf{SL}}\,}}(\frac{n}{2}+1,\mathbb {H})/({{\,\mathrm{\textsf{GL}}\,}}(1,\mathbb {H}){{\,\mathrm{\textsf{SL}}\,}}(\frac{n}{2},\mathbb {H}))$.

We will now prove that Examples 4.3-4.7 provide the full classification of qs-H tiLas. For this step we will use the classification of normal forms of Jordan blocks of the Lie algebras $\mathfrak {sp}(1)$ and $\mathfrak {so}^*(2n)$, presented in [10, Table III]. Next we will denote by $J_{r}(\lambda )$ the standard Jordan block of size *r* corresponding to the eigenvalue $\lambda \in \mathbb {H}$, with the units located under the diagonal, i.e.,

$$ J_{r}(\lambda )=\begin{pmatrix} \lambda &  &  &  \\ 1 & \quad \lambda & \quad & \quad \\ &  \quad \ddots & \quad \ddots &  \quad \\ &  \quad &  \quad 1 & \quad \lambda \end{pmatrix}\,. $$

### Theorem 4.8

There is a bijection between the one-parameter families of isomorphism classes of qs-H tiLas $(\mathfrak {g},\sigma , Q_o, e^{2t}\omega _o)$ $(t\in \mathbb {R})$, and the quaternionic matrices τ presented in Examples 4.3–4.7.

### Proof

For an abelian subalgebra $\mathfrak {t}\subset \mathfrak {so}^*(4n+2)$ we can pick a representative $\tau \in \mathfrak {t}$ of the form (4.1) under the action defined in (4.3), and test whether it satisfies the condition given in (4.2). Recall that τ depends on $a\in \mathfrak {sp}(1)$, $A\in \mathfrak {so}^*(2n)$ and $d\in \mathbb {R}$.

According to [10] and the classification of Jordan blocks for $\mathfrak {so}^*(2n)$, and also for $\mathfrak {sp}(1)$, we can construct the matrix τ by combining the following normal forms:

1. $A\in \mathfrak {so}^*(2n)$ is in block diagonal form with blocks (see cases (29)–(31) in [10, Table III])
  $$ J_{n_u}(0)\,,\qquad J_{n_v}(b_vj)\,, \qquad J_{n_w}(\beta _w)\oplus J_{n_w}(-\beta _w)^*\,, $$
  for $u\in U,v\in V,w\in W$ indexing the blocks, with
  $$ 2n=\sum _{u\in U}n_u+\sum _{v\in V}n_v+2\sum _{w\in W}n_w\,. $$
  Moreover, we have $0<b_v\in \mathbb {R}$ and $\beta _w\in \mathbb {C}$ with $\mathbb{R}\mathbbm{e}(\beta _w)>0,\mathbb{I}\mathbbm{m}(\beta _w)\ge 0$ for $v\in V, w\in W$. Note that some of the cases have two possibilities for $\mathbb {j}$, see cases (30)-(31) in [10, Table III].^1^
2. $a=\mu \cdot i\in \mathfrak {sp}(1)$, for some scalar $\mu \in \mathbb {R}$, and $d\in \mathbb {R}$.

Now, according to the action defined in (4.3) we still have the freedom to rescale *a*, *A*, *d* by $e^{2t} a, e^{2t}A$ and $e^{4t}d$, respectively. Clearly, we can investigate the condition (4.2) for each block separately; Note that a combination of solutions of (4.2) can be glued together to a solution if and only they have the same *a* and *d*.

Applying this procedure, we first observe that in (4.2) only the matrix $A^2$ contributes for order-three nilpotent elements. Thus we can only have Jordan blocks of size $n_u\le 2, n_v\le 2$ and $n_w\le 2$. For order-two nilpotent elements, we see that if $n_u=n_v=n_w=2$ for $u\in U, v\in V,w\in W$, then the conditions

$$ x a=0\,, \ \text {for} \ u\in U\,,\quad 2b_vj x+x a=0\,, \ \text {for} \ v\in V\,, \ \quad 2\beta _w x+x a=0\,, \ \text {for} \ w\in W\,, $$

should be satisfied for all $x\in \mathbb {H}$. Therefore, if we have a=0, then $n_u=2$ is possible and d=0 follows from (4.2), while a direct computation shows that the other cases are not possible, i.e., the cases $n_v=1,n_w=1$ for all v∈V and w∈W.

In the situation when $n_u=n_v=n_w=1$, for $u\in U, v\in V$ and w∈W, by (4.2) we obtain that the conditions

$$\begin{aligned} xd-xa^2=0\,,&\quad \text {for} \ u\in U\,,\\ xd+2b_vjxa-xa^2+(b_v)^2x=0\,,&\quad \text {for} \ v\in V\,, \\ xd+2\beta _w xa-xa^2-\beta _w^2x=0\,,&\quad \text {for} \ w\in W\,, \end{aligned}$$

should be satisfied for all $x\in \mathbb {H}$. Thus here, after rescaling, we deduce that the pairs (a=0,d=0) and (a=i,d=-1) are the only solutions for u∈U, the triple $(a=0,b_v=1,d=-1)$ is the only solution for v∈V and the triple $(a=0,d=1,\beta _w=1)$ is the only solution for w∈W.

All together the above analysis yields the following possible combinations:

(i) a=d=0 and *A* consists of blocks $J_{1}(0)$ and $J_{2}(0)$ and this gives rise to the representative $\tau \in \mathfrak {t}$ described in Examples 4.3 and 4.4, respectively. This is done by comparing the convention for $\mathbb {j}$ in [10] (see the column *K* in Table III of [10], depending on $\kappa =\pm 1$, for the entry (29)), with our convention of $\mathbb {j}$ in the quaternionic-Darboux bases from [7, Sect. A.2]. This means that for κ=1 the conventions are the same but for κ=-1 the two conventions differ by a conjugate transposition. Moreover, note that the parameters *p*, *q* appearing in these two examples, can be understood in the following way:

- The parameter *p* is the number of the Jordan blocks $J_{2}(0)$ satisfying κ=1.
- The parameter *q* is the number of the Jordan blocks $J_{2}(0)$ satisfying κ=-1.

Note that for the second case, the Jordan block $J_{2}(0)$ appears as its conjugate transpose in the quaternionic-Darboux basis.

(ii) a=i,d=-1 and A=0. This case yields the representative $\tau \in \mathfrak {t}$ described in Example 4.5.

(iii) a=0,d=-1 and *A* consists of Jordan blocks of type $J_{1}(j)$. This corresponds to the representative $\tau \in \mathfrak {t}$ described in Example 4.6. In particular, the parameters *p*, *q* are encoded exactly as in case (i), by inspecting entry (30) rather than entry (29) in [10, Table III].

(iv) a=0,d=1 and *A* consists of blocks of type $J_{1}(1)\oplus J_{1}(-1)$. This gives us the representative $\tau \in \mathfrak {t}$ described in Example 4.7.

Since these cases exhaust all admissible combinations, this completes the proof. □

Theorem 1.2 in introduction now follows as a combination of Theorems 3.4 and 4.8.

### Remark 4.9

The restriction $B_{\mathfrak {m}}$ of the Killing form of $\mathfrak {so}^{*}(2n+4)$ to $\mathfrak {m}$ for the qs-H tiLas from Theorem 4.8 is a multiple of the real trace

$$\begin{aligned}&{{\,\textrm{tr}\,}}_{\mathbb {R}}\left( \begin{pmatrix} 0 &  (AX-Xa)^*\mathbb {j}&  0\\ X &  0 &  AX-Xa\\ 0& -X^*\mathbb {j} &  0, \end{pmatrix}\cdot \begin{pmatrix} 0 &  (AY-Ya)^*\mathbb {j}&  0\\ Y &  0 &  AY-Ya\\ 0&  -Y^*\mathbb {j} &  0, \end{pmatrix}\right) \\&=\mathbb{R}\mathbbm{e}\big ((AX-Xa)^*\mathbb {j}Y-X^*\mathbb {j}(AY-Ya)\big )+{{\,\textrm{tr}\,}}_{\mathbb {R}}\big (X(AY-Ya)^*\mathbb {j}-(AX-Xa)Y^*\mathbb {j}\big )\,. \end{aligned}$$

For the Examples 4.3 and 4.4 we have a=0 and $A\in \mathfrak {so}^*(2n)$ is not of full rank. For these cases we see that $B_{\mathfrak {m}}$ is degenerate. Since however $\mathfrak {g}\subset \mathfrak {so}^*(2n+4)$ is a subalgebra of $\mathfrak {so}^*(2n+4)$, the degeneracy of $B_{\mathfrak {m}}$ implies the degeneracy of the Killing form of $\mathfrak {g}$. This gives an alternative explanation of the fact that the symmetric spaces corresponding to these tiLas are *snot* semisimple.

## Homogeneous torsion-free qs-H structures

We will now focus on the following interesting task:

### Question

Is it possible to adapt the construction described in Sect. 4 to obtain non-symmetric homogeneous manifolds admitting an invariant qs-H structure?

Regarding this question, we first point out that from [15, Table 4] it follows that the homogeneous manifolds in our question cannot have full holonomy ${{\,\mathrm{\textsf{SO}}\,}}^*(2n){{\,\mathrm{\textsf{Sp}}\,}}(1)$. Below we will prove that homogeneous almost qs-H manifolds which are torsion-free, i.e., homogeneous qs-H manifolds, are necessarily symmetric spaces, and thus classified by the results in Sect. 4.

Therefore, let us turn our attention again to the construction described in Sect. 4, but this time consider 1-dimensional abelian subalgebras $\mathfrak {t}\subset \mathfrak {so}^*(2n+4)$ generated by general elements $\hat{\tau }\in \mathfrak {so}^*(2n+4)$ of the form

$$ \hat{\tau }=\begin{pmatrix} a &  \quad C^*\mathbb {j}& \quad d\\ 0 &  \quad A & \quad C\\ 1& \quad 0 &  \quad a \end{pmatrix}, $$

with $a\in \mathfrak {sp}(1)$, $A\in \mathfrak {so}^*(2n)$, $d\in \mathbb {R}$ and $C\in \mathbb {H}^n$. Obviously, $\hat{\tau }$ is of the form (4.1) if and only if C=0.

For C≠0 next we will examine when the qs-H structure provided by the construction is a non-symmetric homogeneous qs-H manifold. In this case, we will show that the condition

$$ [\hat{\tau },\mathfrak {m}]\subset \mathfrak {t}, $$

cannot be satisfied for sufficiently large subspaces $\mathfrak {m}\subset \mathfrak {so}^*(2n+4)$, proving the following result, that is, Theorem 1.1.

### Theorem 5.1

A homogeneous qs-H manifold is a qs-H symmetric space.

### Proof

As we want to have elements of $\mathfrak {m}\subset \mathfrak {so}^*(2n+4)$ in each tangent direction, we consider the elements

$$\begin{aligned} \tilde{X}=\begin{pmatrix} \lambda (X)+\mu (X) &  \quad f(X)^*\mathbb {j}& \quad g(X)\\ X & \quad \gamma (X) & \quad f(X)\\ 0& \quad -X^*\mathbb {j} &  \quad -\lambda (X)+\mu (X) \end{pmatrix}\,, \end{aligned}$$

for all $X\in \mathbb {H}^n$ and some linear maps $f : \mathbb {H}^n\rightarrow \mathbb {H}^n$, $g,\lambda : \mathbb {H}^n\rightarrow \mathbb {R}$, $\mu : \mathbb {H}^n\rightarrow \mathfrak {sp}(1)$ and $\gamma : \mathbb {H}^n\rightarrow \mathfrak {so}^*(2n)$. If $[\hat{\tau },\tilde{X}]\subset \mathfrak {t}$ is satisfied for all $X\in \mathbb {H}^n$, that is $\hat{\tau }$ represents a homogeneous qs-H structure, then the 1-form

$$ \lambda +\mu +\gamma : \mathfrak {m}\rightarrow \mathbb {R}\oplus \mathfrak {sp}(1) \oplus \mathfrak {so}^*(2n) $$

should define the connection 1-form of a torsion-free ${{\,\mathrm{\textsf{SO}}\,}}^*(2n){{\,\mathrm{\textsf{Sp}}\,}}(1)$-connection. In particular, λ=0 has to be satisfied as this part is not valued in $\mathfrak {so}^*(2n)\oplus \mathfrak {sp}(1)$. Therefore, in this case the grading component -2 of $[\hat{\tau },\tilde{X}]$ vanishes, and this gives $[\hat{\tau },\tilde{X}]=0$. Moreover, we see that the position (1, 1) of the matrix $[\hat{\tau },\tilde{X}]$ reads as

$$ a\mu (X)+C^*\mathbb {j}X-\mu (X)a-g(X)\,. $$

Since $[\hat{\tau },\tilde{X}]=0$, we thus get the condition

$$\begin{aligned} \mathbb{I}\mathbbm{m}(a\mu (X)+C^*\mathbb {j}X-\mu (X)a-g(X))= &   \frac{1}{2}(C^*\mathbb {j}X+X^*\mathbb {j}C)\\= &   g_1(C,X)i+g_2(C,X)j+g_3(C,X)k\\  = &   0\,, \end{aligned}$$

for all $X\in \mathbb {H}^n$, where $g_{1}, g_2, g_3$ are the pseudo-Hermitian metrics on the linear model $[{{\,\mathrm{\textsf{E}}\,}}{{\,\mathrm{\textsf{H}}\,}}]$ (see [7, Proposition 2.15]). As the metrics $g_1,g_2,g_3$ are non-degenerate, C=0 follows. Therefore, $\hat{\tau }$ is of the form (4.1) and consequently the homogeneous qs-H manifold is a qs-H symmetric space. This completes our proof. □

Let us emphasize that being torsion-free is a key condition in Theorem 5.1. This is because, in general, the output of this construction is a conformally qs-H structure, see [5]. In terms of intrinsic torsion components this means that the almost qs-H structure obtained in this way is of type $\mathcal {X}_4$ (see [8, Remark 1.6]). Let us indeed present an example of a homogeneous almost qs-H manifold with non-trivial torsion lying in the component $\mathcal {X}_4$.

### Example 5.2

Let us consider the element

$$ \hat{\tau }=\begin{pmatrix} i &  -2i+2j &  0 &  3 \\ 0 &  2j &  0 & 2-2k \\ 0 &  0 &  0 & 0 \\ 1 &  0 & 0 &  i \end{pmatrix} $$

with block decomposition of blocks of size 1,1,n-1,1 and set $\mathbb {j}=j.$ Then the subspace $\mathfrak {m}\subset \mathfrak {so}^*(2n+4)$ is given by

$$ \begin{pmatrix} X_1^*(k-1) +2(x_2-x_3)i&  2X_1^* +iX_1^*j &  iX_2^*j &  2(x_2-x_3) \\ X_1 &  0 &  ( i-j)X_2^*j & 2jX_1-X_1i \\ X_2 & - X_2(1+k) &  0 & -X_2i \\ 0 & -X_1^*j &  -X_2^*j & (1+k)X_1 +2(x_2-x_3)i \end{pmatrix}, $$

for $X_1:=x_1+x_2i+x_3j+x_4k\in \mathbb {H}$ and $X_2\in \mathbb {H}^{n-1}$. We can directly check

$$ [\hat{\tau },\mathfrak {m}]=2(x_4-x_1)\hat{\tau }$$

and thus this induces a homogeneous almost qs-H structure, where the torsion belongs to the $\mathcal {X}_4$-component. Recalling that $\mathcal {X}_{4}\cong [{{\,\mathrm{\textsf{E}}\,}}{{\,\mathrm{\textsf{H}}\,}}]$ (see [7]), one can interpret the torsion by the 1-form λ appearing in the proof of Theorem 5.1. This is now given by

$$ \lambda ((x_1+x_2i+x_3j+x_4k,X_2)^{t})=x_4-x_1\,. $$

Obviously, analyzing the corresponding full qs-H automorphism algebra is a non-trivial task. We observe, however, that for n=2 the qs-H automorphism algebra contains a 9-dimensional solvable subalgebra generated by $\mathfrak {m}$.

## Data Availability

Not applicable.

## Footnotes

1 We mention that in [10] the quaternions are represented by 2×2 complex matrices.
